# Cereulide synthetase gene cluster from emetic *Bacillus cereus*: Structure and location on a mega virulence plasmid related to *Bacillus anthracis *toxin plasmid pXO1

**DOI:** 10.1186/1471-2180-6-20

**Published:** 2006-03-02

**Authors:** Monika Ehling-Schulz, Martina Fricker, Harald Grallert, Petra Rieck, Martin Wagner, Siegfried Scherer

**Affiliations:** 1 Lehrstuhl für Mikrobielle Ökologie, Department für Biowissenschaftliche Grundlagen, WZW, Technische Universität München, Freising, Germany; 2 Institute of Milk Hygiene, Milk Technology and Food Science, University of Veterinary Medicine, Vienna, Austria

## Abstract

**Background:**

Cereulide, a depsipeptide structurally related to valinomycin, is responsible for the emetic type of gastrointestinal disease caused by *Bacillus cereus*. Recently, it has been shown that this toxin is produced by a nonribosomal peptide synthetase (NRPS), but its exact genetic organization and biochemical synthesis is unknown.

**Results:**

The complete sequence of the cereulide synthetase (*ces*) gene cluster, which encodes the enzymatic machinery required for the biosynthesis of cereulide, was dissected. The 24 kb *ces *gene cluster comprises 7 CDSs and includes, besides the typical NRPS genes like a phosphopantetheinyl transferase and two CDSs encoding enzyme modules for the activation and incorporation of monomers in the growing peptide chain, a CDS encoding a putative hydrolase in the upstream region and an ABC transporter in the downstream part. The enzyme modules responsible for incorporation of the hydroxyl acids showed an unusual structure while the modules responsible for the activation of the amino acids Ala and Val showed the typical domain organization of NRPS. The *ces *gene locus is flanked by genetic regions with high homology to virulence plasmids of *B. cereus*, *Bacillus thuringiensis *and *Bacillus anthracis*. PFGE and Southern hybridization showed that the *ces *genes are restricted to emetic *B. cereus *and indeed located on a 208 kb megaplasmid, which has high similarities to pXO1-like plasmids.

**Conclusion:**

The *ces *gene cluster that is located on a pXO1-like virulence plasmid represents, beside the insecticidal and the anthrax toxins, a third type of *B. cereus *group toxins encoded on megaplasmids. The *ces *genes are restricted to emetic toxin producers, but pXO1-like plasmids are also present in emetic-like strains. These data might indicate the presence of an ancient plasmid in *B. cereus *which has acquired different virulence genes over time. Due to the unusual structure of the hydroxyl acid incorporating enzyme modules of Ces, substantial biochemical efforts will be required to dissect the complete biochemical pathway of cereulide synthesis.

## Background

*Bacillus cereus *belongs to the *B. cereus *group of organisms that comprises the genetically highly related organisms *Bacillus anthracis*, *B. cereus*, *Bacillus mycoides*, *Bacillus pseudomycoides, Bacillus thuringiensis*, and *Bacillus weihenstephanensis*, (16, 33)which for the most part are capable of producing significantly different toxins. *B. anthracis *causes the fatal animal and human disease anthrax, whereas *B. thuringiensis *produces insecticidal toxins that are commercially used as biocontrol agents [1-3]. Toxin producing *B. cereus *plays an important role as the causative agent of two types of food poisoning: diarrhea and emesis (for review see Granum [4] and Ehling-Schulz *et al*., [5]). The emetic syndrome caused by *B. cereus *is mainly characterized by vomiting a few hours after ingestion of the contaminated food while diarrhoeal poisoning is caused by heat-labile enterotoxins produced during vegetative growth of *B. cereus *in the small intestine [6]. The latter, diarrhea eliciting, toxins are well characterized at the molecular and the transcriptional level [7-10]. Far less is known about the emesis causing toxin cereulide, which has twice been reported to have been involved in the death of a child due to liver failure [11,12].

Cereulide is a small, heat and acid stable cyclic dodecadepsipeptide which is chemically closely related to the potassium ionophore valinomycin [13]. It is toxic to mitochondria by acting as a potassium ionophore (2, 25)and has been reported to inhibit human natural killer cells [14]. According to its chemical structure one could expect cereulide to be synthesized enzymatically via nonribosomal peptide synthetases (NRPSs).

NRPSs are large multifunctional proteins that have a modular organization. The modules, which show a conserved domain structure, selectively catalyze activation and thioester formation of one amino, α-hydroxy or carboxylic acid monomer [15,16]. A minimal module comprises an adenylation (A domain) that activates the cognate substrate and a thiolation domain (T domain) that binds the activated substrate. Chain elongation of the peptide is catalyzed by condensation domains (C domains), located at N-terminal ends of modules which accept acyl groups from the preceding modules [17]. A C-terminal thioesterase domain (TE domain) catalyzes the release of the mature NRPS-bound peptide product [18]. Some modules contain additional domains, like epimerization and methylation domains, that modify the incorporated constituents (for review see Sieber and Marahiel [19]). The order of modules usually corresponds directly to the order of monomers in the peptide product [20,21]. Quite recently, we identified a putative valine activating module that was highly conserved among emetic *B. cereus*. Disruption of the corresponding gene by insertion mutagenesis revealed cereulide deficient mutants, providing for the first time unequivocal evidence for nonribosomal assembly of the emetic toxin cereulide [22].

The main virulence factors of *B. anthracis *and insecticidal toxin genes of *B. thuringiensis *are located on large, well characterized virulence plasmids while diarrhea eliciting enterotoxins of *B. cereus *are located chromosomally. However, the exact genomic location and organization of the cereulide synthetase genes, which represent the main virulence factors of the emetic type of *B. cereus*, was hitherto unknown. The aim of this work was to completely sequence and characterize the genetic locus responsible for cereulide synthesis including the flanking genomic regions in order to gain insight into the synthesis mechanism of this peptide toxin, to determine the genomic location of the *ces *gene cluster and to study its conservation among *B. cereus *group members.

## Results

### Organization of the cereulide synthetase (*ces*) gene cluster

**Figure 1:**
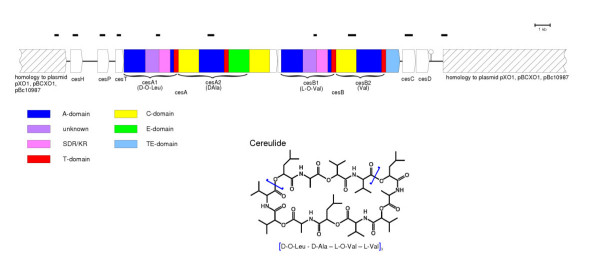
**Biosynthetic gene cluster for cereulide synthesis**. The domain organization of the structural cereulide synthetase genes *cesA *and *cesB *is indicated (see Results for details) and the flanking regions showing homologies to toxin plasmids from *B. cereus *group members are printed as hatched boxes. For details on CDS designation see Table 2. The bars refer to probes used to test the conservation of *ces *genes in the *B. cereus *group (see Table 3) Inset: Structure of cereulide according to [13].

The total sequence of the cereulide synthetase gene cluster (24 kb) was dissected by module jumping and inverse PCR. Sequence analysis showed that the *ces *gene cluster encodes 7 CDSs: *cesH*, *cesP, cesT*, *cesA, cesB*, *cesC *and *cesD *(Fig. 1). CesH is located at the 5' end of the cluster 990 bp upstream of *cesP *in the same reading frame. *cesT *is located 268 bp downstream from *cesP *while *cesA *overlaps with *cesT*. *cesB *is separated by 13 bp from the stop codon of *cesA. cesC *and *cesD *are located in the 3' part of the *ces *gene cluster (Fig. 1). No termination structures were predicted, neither between the CDSs *cesPTABCD *nor between *cesH *and *cesP*, but downstream of the *cesD *gene a hairpin structure was predicted using Mfold [23], indicative of the presence of a terminator sequence. The deduced protein sequence (Table 2) from *cesH *(31 kDa) showed significant (58%) identity to putative hydrolases/acyltransferases (COG0596) from *B. cereus *group members. The following CDS, designated *cesP *(28.9 kDa), showed high homology (32–38% identity and approx. 60% similarity) to the 4'-phosphopantetheinyl transferase from *Bacillus brevis *and *Bacillus subtilis *involved in nonribosomal synthesis of gramicidin S (Gsp; Swissprot database accession no. P40683) and to surfactin (Sfp; Swissprot database accession no. P39135), respectively [24,25]. *cesT *starts with a GTG and is preceded by a putative RBS (AGGAG) 5 bp upstream of the start codon. CesT (27.6 kDa) showed significant similarity to BacT from *Bacillus licheniformis *(GenBank accession no. AF007865.2; 33% identity and 56% similarity), GrsT from *B. brevis *(GenBank accession no. AJ566197; 35% identity, 53% similarity) and other thioesterases associated with NRPS. The structural genes of cereulide synthetase are coded by the two large CDSs (10 kb and 8 kb) *cesA *and *cesB*. CesA and CesB, each of which is responsible for the activation and incorporation of two monomers in the peptide chain, show high homology to known NRPS and PKS-NRPS hybrids. Alignment of CesA and CesB revealed 32% identity and 53% similarity between these two proteins. *cesC *and *cesD*, located in the 3' part of the *ces *gene cluster, encode a putative ABC transporter. ABC binding cassettes and transmembrane domains, typical for the ABC transporter family, were identified in these sequences.

### Analysis of the NRPS domains of cereulide synthetases

**Figure 2:**
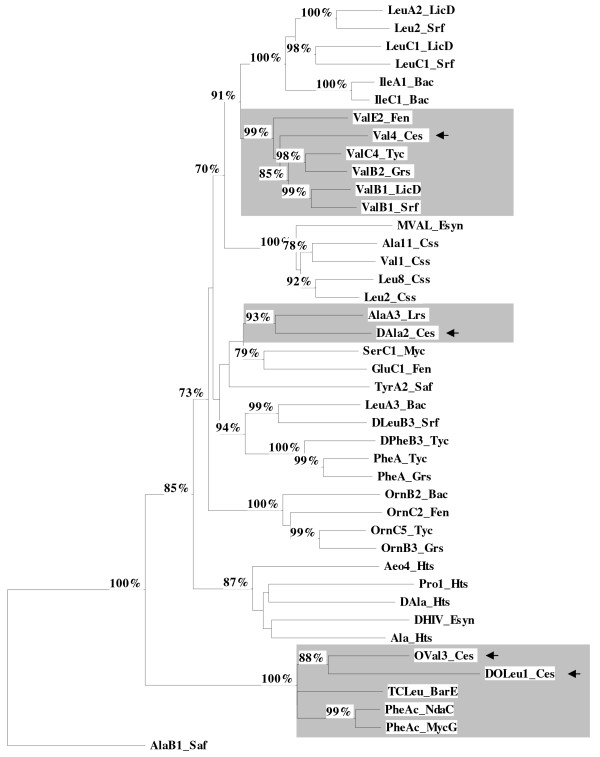
**Genetic analysis of A domains**. (A3 motif to the A6 motif) from nonribosomal peptide synthetases. The tree was constructed with TREECON [48] using the neighbor-joining method. All bootstrap values > 70% (1000 replicates) are shown next to the nodes. A domains from the cereulide synthetase (Ces) are printed in bold type. Abbreviations: Bac: bacitracin synthetase; Bar: barbamide synthetase; Css: cyclosporine synthetase; Esyn: Enniatin; Fen: fengycin synthetase; Grs: gramicidin S synthetase; Hts: HC-toxin; LicD: lichenysin synthetase; Myc: mycosubtilin synthetase; Nda: nodularin synthetase; Saf: Saframycin synthetase; Srf: surfactin synthetase; Tyc: tyrocidine A synthetase.

**Figure 3:**
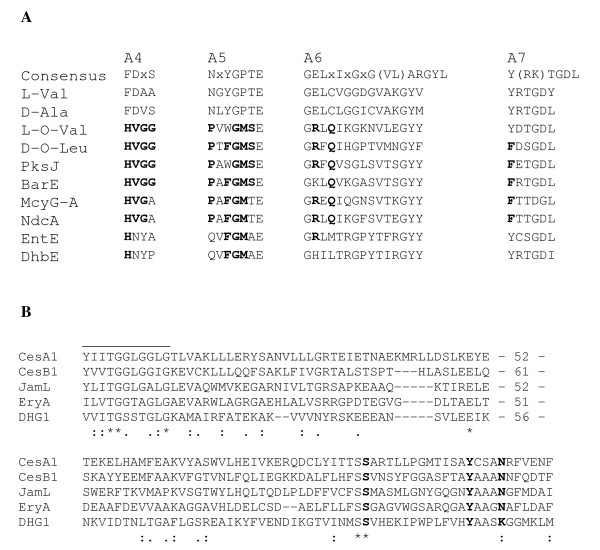
**(A) Alignment of the adenylation**. **(A) domain core motifs lining the substrate binding pocket **of Ces and unusual core motifs from other bacterial NRPS. Consensus sequence of core motifs A4 to A7 (according to [53]) is depicted. Residues identical to amino acids from Ces core motifs are printed in boldface type. **(B) Insertions in A domains **from CesA2 (D-*O-*Leu) and CesB1 (L-*O*-Val) were aligned to short chain dehydrogenases (SDR) and ketoreductases (KR); partial sequences including putative NADPH binding sites (solid bar) and the catalytic residues of SDRs/KR (printed in boldface type). Numbers between hyphens in the first lines refer to residues not shown before residues depicted in the second lines. JamL: KR from Jamaicamides synthetase (GenBank accession no. AY522504) of *Lyngbya majuscula*; EryA: KR from erythronolide synthetase of *Saccharopolyspora erythraea *(GenBank accession no. M63676). GlcDh: Glucose 1-dehydrogenase from *B. megaterium *(Swissprot accession no. P39482).

The CesA synthetase harbors two modules with the domain structure A_i_-x-A_ii_-T-C-A-T-E-C and the CesB synthetase shows the domain structure A_i_-x-A_ii_-T-C-A-T-TE (Fig. 1). A_i _comprises the conserved core motifs A1–A8 while A_ii _contains the core motifs A9 and A10, and x refers to a region of unknown function. Generally, the order of modules in a given NRPS correlates to the order of monomers in the peptide product, hence CesA is expected to activate the precursors for D-*O*-Leu (CesA1) and D-Ala (CesA2), while CesB is expected to be involved in the activation of a precursor of L-*O*-Val (CesB1) and the activation and incorporation of the proteinogenic amino acid L-Val (CesB2). Using the amino acid specificity code [26], the substrate activated by the modules CesA2 and CesB2 could be clearly predicted to be Ala and Val, while no A domain specificities could be predicted for the modules CesA1 and CesB1 (data not shown). However, alignment and genetic analysis of A domains revealed significant similarities between the latter modules, which are involved in the incorporation of the non-proteinogenic oxy acids (Fig. 2). A comparison of the A domain core motifs showed that these are less conserved in CesA1 and CesB1 than in CesA2 and CesB2 (Fig. 3A). In addition, the A domains of CesA1 and CesB1 are interrupted by an insertion (x) of about 550 amino acids between the A8 and A9 core motifs. The inserted sequences from CesA1 and CesB1 showed 25% identity and 40% similarity. Comparison to database sequences revealed no significant homology of the first 320 aa of these sequences, while the C-terminal part of the insertions showed significant similarities to enzymes belonging to the short chain dehydrogenase/reductase (SDR) superfamily (Fig. 3B). The module CesA2 contains an epimerase (E) domain, which is consistent with the D-stereochemistry of the Ala residue of cereulide. In the C-terminal region of *CesB *a putative thioesterase (TE) domain comprising 254 amino acid residues (29 kDa) was identified (Table 2).

### Sequence and analysis of flanking regions of the *ces *operon

The flanking regions of the *ces *operon were sequenced by inverse PCR and the resulting sequences were searched against NCBI's non-redundant database using BLAST algorithms. The 5' region and the 3' region of the *ces *genes showed significant homology (about 90% identity) to *B. anthracis *toxin-encoding pXO1 plasmid sequences and to pBc10987 [27]. To gain additional sequence information primers were designed using *B. anthracis *plasmid pXO1 sequence information. A total of 3.5 kb upstream and 9.5 kb downstream of the *ces *operon was sequenced. The corresponding upstream CDSs showed high identities (60% to 98%) to hypothetical proteins located on virulence plasmids of *B. anthracis*, *B. thuringiensis*, and *B. cereus *(pXO1 (GenBank Acc. no. AE017336), pBtoxis (GenBank Acc. no. AL731825), pE33L9 (GenBank Acc. no. CP000044), pBCXO1 (GenBank Acc. no. AAEK01000000) and pBc10987 (GenBank Acc. no. AE017195)). Sequence analysis of the downstream region revealed 7 hypothetical proteins with homologies of more than 90% to hypothetical proteins from pXO1, pBCXO1 and pBc10987. The region downstream of the *ces *genes is nearly identical to the corresponding sequence of pBc10987 (99% identity), while no significant homology to pBtoxis could be observed. The sequence of the DNA region upstream from *ces *is 94% identical to the corresponding sequence of pBc10987 and 88% identical to pBtoxis, respectively.

### PFGE and hybridization studies

**Figure 4:**
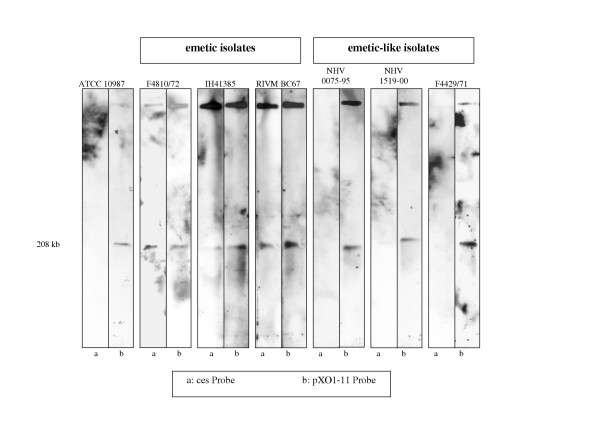
**Hybridization of emetic and emetic-like *B. cereus *with probes targeting the *ces *operon (a) and a pXO1 related CDS (b)**. Total DNA was separated by PFGE, transferred to a membrane and hybridized with a *cesB *specific probe and a probe derived from pXO1-11 (for details on probes see Supplemental Materials Table S1). Hybridization with both probes revealed a single band for emetic strains which has the same size as the pBc10987 plasmid from *B. cereus *ATCC 10987 (lane 2).

Undigested genomic DNA from selected emetic and emetic-like *B. cereus *strains was separated by PFGE, blotted on a membrane and hybridized to probes that were directed either against the cereulide synthetase genes (*cesB *probe obtained from emetic *B. cereus*) or against a CDS located in the flanking region of the *ces *genes (pXO1-11 probe obtained from *B. anthracis*). The hybridization results (see Fig. 4) showed that the cereulide synthetase is located on a megaplasmid, designated pBCE4810, of the same size as pBc10987 (208 kb). No hybridization signals were obtained from the non-emetic strains tested. Emetic-like strains reacted positively with the pXO1-11 derived probe but not with the *ces *probe. However, the sizes of the plasmids reacting with pXO1-11 differed slightly from that of the emetic strains (Fig. 4). Additional probes targeting different modules of the cereulide synthetase operon and its flanking regions were designed, and slot blot analysis was carried out to determine if parts of the *ces *gene locus are conserved in non-emetic *B. cereus *group strains. None of the non-emetic strains reacted with any of the *ces *genes derived probes (Table 3), while some of the strains, especially emetic-like strains, were positive in hybridization assays with pXO1 derived probes (Table 4). The corresponding plasmid from one of these emetic-like strains, pBCEL1519 from *B. cereus *NVH 1519-00, was partially sequenced using primers designed from pBCE4810 sequence with similarity to pXO1.

## Discussion

### The cereulide synthetase operon

We have sequenced a 36 kb genetic region in the *B. cereus *emetic reference strain F4810/72 involved in the biosynthesis of cereulide. Previous knock out mutants have shown that this toxin is produced by a multi-enzyme complex named cereulide synthetase [22]. The chemical structure of cereulide is reflected in the genetic organization of its synthetase genes (Fig. 1). This gene cluster comprises, besides the typical genes such as a 4'-phosphopantetheinyl transferase (*cesP*) essential for activating the NRPS (apoenzyme to holoenzyme) and the structural genes responsible for the assembly of the peptide product, a putative type II thioesterase (*cesT*) which could potentially remove misprimed monomers and regenerate the NRPS [28]. A putative hydrolase (c*esH*) is located in the 5' part of the cereulide biosynthesis cluster and a putative ABC transporter (c*esC/D*) is encoded in its 3' part. CesC/D might either be involved in the transport of cereulide or confer self resistance towards cereulide.

The structure of the *ces *genes and number of modules predicts a monomeric tetrapeptide but cereulide is a trimeric depsipeptide (Fig. 1). A similar structure has been described for gramicidin S and enterobactin. Synthesis of those peptides is carried out by one NRPS that is regenerated and repetitively used for synthesis. In case of such iterative processes the TE domains have additional functions allowing the collinear synthesis to be repeated two or three times (for review see [19]). The exact mechanism of these iterative TEs is not yet known, but mutation studies provided evidence that the TE domain of *E. coli *EntF catalyzes cyclolactonization as well as chain elongation [29]. A similar mechanism resulting in intramolecular trimerization might also be involved in cereulide assembly.

### Unusual structure of heterocompound activating modules

The A domains of the heterocompound activating modules CesA1 and CesB1 are less conserved than the A domains from CesA2 and CesB2, which activate the amino acid moieties (Fig. 2). Especially, the core motifs A4 and A5 differ significantly from the typical consensus motifs described by Marahiel *et al*., [20]. Nevertheless, they show some homology to the corresponding motifs of phenylacetate activating modules MycG-A and NdaC and to aryl acid activating modules DhbE and EntE [30,31] (see Fig. 3A). The crystal structures of the phenylalanine-activating A domain of gramicidin S synthetase and the structure of DhbE involved in bacillibactin synthesis revealed a decisive role of the core motifs A4 and A5 in substrate binding and selection [31,32]. The altered A4 and A5 core motifs found in CesA1 and CesB1 did not allow substrate prediction for these modules. The A4 motif usually represents the first anchor in amino acid activating domains, however this core motif is missing in DhbE as its substrate does not contain any α-amino-group. In DhbE the A4 motif, as well as the A5, is replaced by another sequence motif that is thought to be specific for carboxy acid activating enzymes [31] while the residue K517 is conserved in α-amino acid activating domains, in the carboy activating domain of DhbE, as well as in CesA1 and CesB1 (data not shown). Since the A4 and A5 motifs of the two hydroxy acid incorporating modules of the cereulide synthetase are quite similar, it is tempting to speculate that the motifs are characteristic for a special type of substrate (see Fig. 3A). Detailed biochemical and structural analysis will be necessary to clarify the substrate specificity of these modified A domains. Nevertheless, the altered motifs found in CesA1, CesB1 and other NRPSs might be useful to identify yet unknown A domains activating new types of substrates.

In addition, about 550 amino acids are inserted between the core motifs A8 and A9 in CesA1 and CesB1 (Fig. 1). Different types of insertions in A domains located at this position have been described for bacterial NRPS such as methyltransferases and oxidoreductases [33,34]. Since the N terminal part of the insertion (x) in CesA1 and CesB1 did not reveal any significant homologies to characterized proteins from database entries, no decisive function could be attributed to these domains in the cereulide synthetase. However, the C terminal part of both insertions showed homologies to short chain dehydrogenases (SDR) and ketoreductases (KR) (Fig. 3B). In addition to the Rossmann fold motif, SDR catalytic Tyr and Ser residues, which are also highly conserved in reductase domains from polyketide synthetases (KR domains) [35], were identified (Fig 3B). As in KR domains the catalytic Lys residue of SDRs is substituted by an Asn in CesA1 and CesB1. It is therefore tempting to speculate that the inserted domains x (KR) might indeed catalyze the ketoreduction of the substrates bound to CesA1 and CesB1, respectively.

### The megaplasmid encoded cereulide synthetase genes are restricted to emetic strains

**Figure 5:**
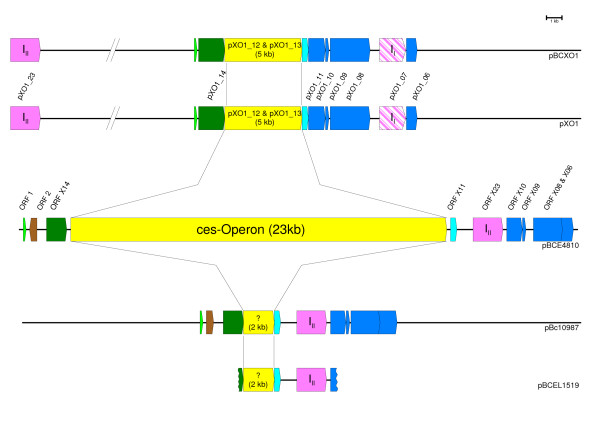
**Comparison of partial sequenced toxin plasmid pBCE4810 to toxin plasmids from *B. cereus *group members**. The genetic region responsible for cereulide production in emetic strains was compared to the *B. anthracis *toxin-encoding plasmid pXO1, to pBCXO1 from *B. cereus *G9241 capable of causing an anthrax-like illness, to pBc10987 from *B. cereus *ATCC 10987 and to pBCEL1519 from the emetic-like strain NVH1519-00. For CDS designation of pBCE and pBCEL see Table 2. I_I_: Group I intron; I_II_: Group II intron.

In order to investigate the distribution and conservation of the *ces *genes among *B. cereus *group members, Southern blot analysis was performed using different probes that target all CDSs belonging to the *ces *operon and hypothetical CDSs located in the periphery of these genes. Special emphasis was placed on emetic-like strains since these are closely related to emetic strains but do not produce cereulide [36]. Our results revealed a strict confinement of *ces *genes to cereulide producers (Table 3). The putative hydrolase encoded by *ces*H was not detected in the non-emetic *B. cereus *group strains and is therefore considered an integral part of the *ces *gene locus. However, several non-emetic strains, especially emetic-like strains, were positive in hybridization studies using probes derived from *ces *flanking pXO1 homolog CDSs (Table 4). A high conservation of pXO1 genes among *B. cereus *group members and other closely related bacilli has previously been reported [37], but no information on emetic or emetic-like strains was provided in this study. Recently it has been reported that cereulide production depends on the presence of a plasmid. Cured emetic strains lost their ability to produce cereulide [38]. Our hybridization experiments (Fig. 4) showed that the cereulide synthetase encoding plasmid pBCE4810 is of the same size as the sequenced pXO1 related plasmid pBc10987 (208 kb) from *B. cereus *ATCC 10987 [27]. The latter, however, does not carry the *ces *genes. Plasmid sizes from emetic-like strains were slightly variable, but partial sequences of the plasmid pBCEL1519 (7.4 kb) from the emetic-like strain NVH1590-00 revealed an identity of nearly 100% to pBc10987 and 90% to pXO1. A comparison of virulence plasmids from *B. cereus *group members and the partially sequenced plasmids pBCE4810 from the emetic *B. cereus *reference strain F4810/72, and pBCEL1519 from the emetic-like strain NVH1519-00, showed that the *ces *gene locus is inserted in a highly conserved part of these virulence plasmids between genes with similarities to pXO1-14 and pXO1-11 (Fig. 5). However, the inserts differ: at this location on pXO1 and pBCXO1 are the genes for pXO1-13, a hypothetical virulence factor, and pXO1-12, whereas pBCE4810 contains the *ces *locus responsible for cereulide synthesis, and pBc10987 and pBCEL1519 both possess an identical 2 kb insert of unknown function. Downstream of this genetic locus a group II intron has recently been described in pBc10987 [39] that is also present in pBCE4810 and pBCEL1519 (Fig. 5). At the corresponding genetic region in pXO1 a group I intron has been identified [39]. Group I and group II introns were also found in other sequenced *B. cereus *group strains. Quite recently, evidence was provided that the group II introns splice in vivo [39]. This ability to splice is a prerequisite for mobility and insertion into new DNA target sites, and could become beneficial for the organism under stressful conditions.

### Evolution of different toxin plasmids from an ancient virulence plasmid in a *B. cereus *ancestor?

The key role of the toxin plasmid pXO1 in anthrax pathogenesis and the importance of toxin harboring plasmids for insecticidal pathogenesis of *B. thuringiensis *are well known, whereas knowledge about the function of *B. cereus *plasmids is quite limited (for review see Rasko *et al*., [40]). Our present work revealed a third type of *B. cereus *group toxins being encoded by a megaplasmid: the biosynthetical genes responsible for the emetic type of *B. cereus *food borne disease are also located on a pXO1-like plasmid (Fig. 4). Thus, all species specific toxins of the *B. cereus *group are located on megaplasmids while the enterotoxins, which are broadly distributed among *B. cereus *group members [41], are localized on the chromosome. BLAST searches using the partial pBCE4810 sequence revealed high similarities (85–99%) of the *ces *gene flanking regions to pXO1 and the *B. cereus *plasmids pBc10987 and the pXO1-like plasmid pBCXO1, and high similarities (87–93%) in the 5' region of *ces *to the insecticidal toxin plasmid pBtoxis and a plasmid designated pE33L9 from a *B. cereus *strain isolated from a dead zebra. Comparison of selected pXO1-like CDSs from F4810/72 revealed an overall identity of about 95% of BCE4810 to pBc10987, about 92% identity to pBCXO1, and about 90% identity to pXO1. Like pBc10987, emetic strains seem to lack the *B. anthracis *PAI encoded virulence genes since no hybridization signals were obtained with probes targeting these genes (Table 4). The data presented in this work will contribute to developing a better understanding of the evolution and distribution of this group of plasmids. One might speculate that a plasmid in an ancestral form has been present in *B. cereus *group strains, which over time has acquired virulence genes conveying different disease causing phenotypes. Nevertheless, a detailed phylogenetic analysis of plasmids-focusing on mobile elements- from all members of the *B. cereus *group will be necessary to unravel the role of plasmids in pathogenesis and evolution of the *B. cereus *group of organisms.

## Conclusion

The characterization of the *ces *genes illustrated the high flexibility of the module organization of bacterial NRPS. A new type of insertion in A domains has been observed in the modules CesA1 and CesB1. No clear function could be assigned to the inserted domains, although hundreds of NRPS modules have been sequenced and biochemically characterized. The characterization of the cereulide synthetase genes and its flanking regions revealed the extra-chromosomal location of the main virulence factor of emetic *B. cereus *on a plasmid with a pXO1-like backbone. Sequencing of the entire plasmids from emetic and emetic-like strains could provide new insight into the evolution of toxin producing members of the *B. cereus *group, especially elucidation of the evolution of *B. anthracis *and the emetic lineage of *B. cereus*.

## Methods

### Bacterial strains

The cereulide producing reference strain F4810/72 [42] was used to determine the sequence of the complete cereulide synthetase locus and the sequence of flanking regions. Cells were grown on plate count (PC) agar plates or in LB (Luria-Bertani) medium at 30°C. *Escherichia coli *strains used for subcloning were grown at 37°C in LB medium with the appropriate antibiotics. Details on the origin of *B. cereus *group strains used to test the occurrence of the *ces *genes in hybridization studies are provided in Table S1 (see additional files).

### Sequencing strategies

Due to stability problems with clones from a cosmid library, the sequence of the total cereulide synthetase operon and its flanking regions was determined by inverse PCR and module jumping as described previously [22]. The latter sequencing strategy took advantage of highly conserved core motifs in NRPS genes which have been successfully used to design degenerated primers and amplify novel NRPS modules [20,43]. With the combination of a specific primer located in the known sequence and a degenerated primer located in a core motif in a putative flanking module, additional sequence information can be gained by "jumping" from one module to the next one, see e.g. [22]. Organization of the *ces *gene cluster was confirmed by direct sequencing of the antisense strand from genomic DNA. In addition, primers derived from genomic sequences of the *B. anthracis *pXO1 toxin plasmid and the *B. cereus *ATCC 10987 pBc10987 plasmid were used to obtain sequence information from flanking regions of the cereulide synthetase operon. DNA was prepared using the AquaPure Genomic DNA Isolation-Kit (Bio-Rad, Germany). For inverse PCR total chromosomal DNA was isolated by phenol-chloroform extraction as described previously [43]. Preparation of plasmid DNA was performed according to standard procedures [44]. Sequences of degenerated primers successfully used for module jumping are listed in Table 1. Amplicons from module jumping were subcloned in TOPO TA vectors according to the manufacturer's recommendation (Invitrogen, USA) and sequenced as described previously [22]. PCR amplification products obtained with primers predicted from plasmids of *B. cereus *group members and PCR products obtained by inverse PCR were either subcloned in TOPO TA or directly sequenced by using a BigDye Terminator v1.1 Cycle Sequencing kit (Applied Biosystems).

### Sequence analysis

The sequencing analysis software package Vector NTI (Informax Inc., U.S.A.) was used to generate the contig sequence from the sequenced PCR products obtained by PCR, inverse PCR and module jumping and the resulting sequence was searched against the sequenced genomes of *B. cereus *group members. Sequence similarity searches were performed using the Basic Local Aligment Search Tools BLASTX and BLASTP on the NCBI website [45,46]. The software packages ClustalX and TREECON were used for sequence alignments and cluster analysis [47,48]. Substrate specificity of *ces *genes was assessed according to Stachelhaus *et al*; [26]. The terminator search tool [49] available at Heidelberg Unix Sequence Analysis Resources [50] and Mfold [51], available at the Macfarlane Burnet Center's internet site [51], were used for the prediction of termination sequences.

### Hybridization assays

For hybridization studies chromosomal DNA was isolated with the Puregene DNA isolation kit (Gentra, USA) according to the manufacturer's instruction and blotted onto nitrocellulose using a Milliblot-S slot blot manifold (Millipore, USA). Hybridization was performed using digoxigenin-labelled probes (Roche) directed against the different modules of *ces*, genes flanking the *ces *locus and selected pXO1 CDSs. The probes were obtained from the emetic reference strain F4810/72 and *B. anthracis *CIPA2 (provided by Gilles Vernaud, Orsay, France) and *B. anthracis *6–87 (provided by Ulrich Busch, Oberschleissheim, Germany) by PCR using the oligonucleotide primers described in Table S2 (see additional files). Optimal hybridization temperature for each probe was calculated and hybridization was carried out according to recommendations of the manufacturer (Roche, Germany). After hybridization, membranes were washed twice for 15 min in 2 × SSC containing 0.1% SDS at room temperature and two times 15 min in 0.1 × SSC containing 0.1% SDS at 65°C. Detection was performed by enzyme immunoassay according to recommendation of manufacture (Roche, Germany) with the chemiluminescence CDP-Star AP substrate (Novagen, USA).

### Pulse field gel electrophoresis (PFGE)

Preparation of total genomic DNA in agarose plugs was performed as described by Kolsto *et al*., [52]. Electrophoresis was performed using a CHEF DR III System (Bio-Rad) with a 0.8% SeaKem Gold Agarose gel (Cambrex Bio Science, USA) in 0.25 × TBE buffer (25 mM Tris-borate buffer pH 8, 0.05 mM EDTA) at 15°C with a pulse of 5–200 s for 20 hours. Size of the fragments was estimated using lambda concatamers (Bio-Rad) and Salmonella Braenderup Global Standard (PulseNet) H9812 (XbaI digested). After electrophoresis, gels were stained in 100 μg/ml ethidium bromide for 30 min and destained in water for 1 h. Gels were denaturated in 0.25 M HCl for 15 min, followed by soaking the gel in 1.5 M NaCl/0.5 N NaOH with constant agitation for 2 × 30 min. Neutralization was performed in 0.5 M Tris (pH 8)/1.5 M NaCl for 2 × 30 min. Gels were blotted overnight by capillary transfer in 20 × SSC onto Hybond-N+ nitrocellulose membrane (Amersham Biosciences, UK). After blotting, the membranes were rinsed in 6 × SSC, air dried for 30 min at room temperature and finally baked for 2 h at 80°C. Hybridization was performed as described above.

### Nucleotide sequence accession number

The nucleotide sequence from pBCE4810 of *B. cereus *F4810/72 described in this paper has been submitted to GenBank under accession no. DQ360825.

## Authors' contributions

MES was in charge of sequencing and data analysis, conceived the study and did the writing. MF was in charge of the hybridization studies, did part of the sequencing and participated in writing the method section. HG worked at the project as diploma student, doing part of the sequencing and gene cluster analysis. PR and MW performed the PFGE experiments. SS supported the study, participated in its design and contributed to writing.

## Supplementary Material

Additional File 1 Table S1: Origin of *B. cereus *group strains used for hybridization studies Click here for file

Additional File 2 Table S2: Oligonucleotide primers used for southern hybridization and sequencing Click here for file

## Figures and Tables

**Table 1:** Oligonucleotide primers used for module jumping and amplifications

**Primer**	**Sequence (5'-3')**	**Use**
F_C3a	GCA(CT)CA(CT)AT(ACT)AT(ACT)TC(AGCT)GA(CT)GG(AGCT)TGG	Module jumping (C-A domain)
R_T1a	C(AGT)A(GT)(AGT)A(AG)(AT)GA(AG)TG(ACT)CC(AC)CC	Module jumping (A-T domain)
F_A3	GG(ACT)(AT)C(AGT)AC(ACT)GG(ACT)(AC)A(AGCT)CC(ACT)AA(AG)GG	Module jumping (A-T domain)
PL10987_R2	CCGTTATCAGCAATAGTCCTA	Primer derived from pBc10987
PL10987_R4	GAATATACTCAACAGTAGCTACC	Primer derived from pBc10987
PL10987_R5	CGATACGTCAAATGTACTCGG	Primer derived from pBc10987
PL10987_R6	GTGATTCATATTGTCTGGATACG	Primer derived from pBc10987
PL10987_F20	GAAGAGAGATATGTCTGTACT	Primer derived from pBc10987
PL10987_F21	GTATATTGTGGCAATTGATGAAGC	Primer derived from pBc10987

a) see reference [22]. Primers for hybridization studies are provided in the supplement (see additional file Table S1); primers for inverse PCR and control sequence reaction are not shown

**Table 2:** Deduced function of encoded proteins in the *ces *operon and flanking regions

Protein^a^	Length of product (aa)	Deduced function (Homology)
CDS 1	63	Hypothetical protein (pXO1 related)
CDS 2	159	CAAX amino terminal protease
CDS X14	565	Conserved protein (Clp ATPase^b^) (pXO1-14 like)
*Ces *gene cluster		Cereulide biosynthesis
CesH	269	Hydrolase/Acyltransferase
CesP	251	Phosphopantheteinyltransferase
CesT	237	Type II Thioesterase
CesA	3391	NRPS (Cereulide synthesis)
CesB	2681	NRPS (Cereulide synthesis)
CesC	291	ABC transporter
CesD	268	ABC transporter, Permease
CDS X11	137	Hypothetical protein (pXO1-11 like)
CDS X23	642	Group II Intron (Reverse transcriptase, maturase)
CDS X10	333	Hypothetical protein (Methyltransferase^b^) (pXO1-10 like)

a) CDS designation: CDSs with homolgies to pXO1 CDSs are indicated by "X" and the corresponding CDS number of pXO1; CDSs belonging to the *ces *gene cluster are named *ces*; b) Putative function as determined by Ariel *et al*., [54].

**Table 3:** Occurrence of *ces *genes in *B. cereus *group members tested by hybridization assays. Several probes targeting all CDSs of the *ces *operon and CDSs from flanking regions (see Table 4) were used to test the distribution of *ces *genes in the *B. cereus *group.

**Strain^b^**	**Subtype^a^**	**cesH**	**cesP**	**cesT**	**cesA1**	**cesA2**	**cesB1**	**cesB2**	**cesC**
*B. cereus*									
F4810/72	E	x^c^	x	x	x	x	x	x	x
MHI 1305	E	x	x	x	x	x	x	x	x
UHDAM IH41385	E	x	x	x	x	x	x	x	x
NVH 0075-95	EL	-	-	-	-	-	-	-	(x)
NVH 1519-00	EL	-	-	-	-	-	-	-	-
INRA C24	EL	-	-	-	-	-	-	-	-
F3003/73	EL	-	-	-	-	-	-	-	-
F4429/71	EL	-	-	-	-	-	-	-	-
NVH 200	EL	(x)	-	-	-	-	-	-	-
RIVM-BC-0063	EL	-	-	-	-	-	-	-	-
ATCC 10987	EL	-	-	-	-	-	-	-	-
WSBC 10892	EL	-	-	-	-	-	-	-	-
WSBC 10028	NE	-	-	-	-	-	-	(x)	(x)
WSBC 10035	NE	-	-	-	-	-	-	-	-
ATCC 14579^T^	NE							-	
*B. thuringiensis*									
WS 2620	NE	-	-	-	-	-	-	-	-
WS 2621	NE	-	-	-	-	-	-	-	-
WS 2632	NE	-	-	-	-	-	-	-	-
WSBC 28001	NE	-	-	-	-	-	-	-	-
WSBC 28002	NE	-	-	-	-	-	-	-	-
WSBC 28022	NE	-	-	-	-	-	-		-
WSBC 28023	NE	-	-	-	-	-	-		-
WSBC 28024	NE	-	-	-	-	-	-		-
*B. mycoides*									
WSBC 10256	NE	-	-	-	-	-	-	-	-
WSBC 10257	NE	-	-	-	-	-	-		-
WSBC 10258	NE	-	-	-	-	-	-		-
WSBC 10276	NE	-	-	-	-	-	-		-
WSBC 10278	NE	-	-	-	-	-	-		-
WSBC 10292	NE	-	-	-	-	-	-		-
WSBC 10293	NE	-	-	-	-	-	-	-	-
WSBC 10360	NE	-	-	-	-	-	-	-	-
*B. weihenstephanensis*									
WSBC 10001	NE	-	-	-	-	-	-		-
WSBC 10045	NE	-	-	-	-	-	-		-
WSBC 10202	NE	-	-	-	-	-	-		-
WSBC 10204^T^	NE	-	-	(x)	-	-	-	-	-
WSBC 10212	NE	-	-	-	-	-	-		-
WSBC 10296	NE	-	-	-	-	-	-		-

a) *B. cereus *group members are classified as emetic (E), emetic-like (EL) and non-emetic (NE) according to [36]. b) for strain designation and details on strains tested see additional file Table S1; c) X hybridization observed; (X) weak hybridization signal; – no hybridization signal.

**Table 4:** Conservation of pXO1 sequence in the emetic lineage of *B. cereus*.

**Strain**	**Subgroup^a^**	**pXO1-11**	**pXO1-14**	**pXO1-23**	**pXO1-55**	**pXO1-98**	**pXO1-lef**	**pXO1-pagA**	**pXO1-atxA**	**pXO1-136 cot43**	**pXO1-142**
F4810/72	E	x^b^	x	x	x	x	-	-	-	x	x
MHI 1305	E	x	x	x	x	x	-	-	(x)	x	x
UHDAM-IH41385	E	x	x	x	x	-	-	-	-	x	x
NVH 0075-95	EL	x	x	x	x	-	-	-	-	-	x
NVH 1519-00	EL	x	x	x	x	x	-	-	-	-	x
F4429/71	EL	-	x	x	x	-	-	-	-	(x)	-
NVH 200	EL	-	-	x	x	-	-	-	-	x	x
RIVM-BC-0063	EL	-	x	x	(x)	x	-	-	-	-	-
WSBC 10892	EL	x	-	x	-	x	-	-	-	x	x
WSBC 10028	NE	-	-	-	(x)	-	-	-	-	-	(x)
WSBC 10035	NE	-	(x)	x	(x)	-	-	-	-	-	-
WSBC 10204^T^	NE	-	(x)	-.	x	-	-	-	-	-	-
ATCC 14579^T^	NE	-	x	-	x	-	-	-	-	-	-

a) E: emetic strains and EL: emetic-like strains as defined by Ehling-Schulz *et al*., [36]; NE refers to non-emetic strains. b) X hybridization observed; (X) weak hybridization signal; – no hybridization signal.
