# Supplementary material for: Cereulide synthetase gene cluster from emetic Bacillus cereus: Structure and location on a mega virulence plasmid related to Bacillus anthracis toxin plasmid pXO1
Source: BMC Microbiol. 2006 Mar 2;6:20. doi: 10.1186/1471-2180-6-20 (PMC1459170; doi:10.1186/1471-2180-6-20)
Supplement: Additional File 1 — Table S1: Origin of B. cereus group strains used for hybridization studies [file 1471-2180-6-20-S1.doc]

## Table S1: Origin of *B. cereus* group strains used for hybridization studies.

| **Species and strain^a^** | **Characteristic** | **Origin** |
| --- | --- | --- |
| *Bacillus cereus* | | |
| F4810/72 (SMR-178) | Emetic | Vomit (patient) |
| MHI 1305 | Emetic | Food remnants associated with emetic outbreaks |
| UHDAM IH41385 | Emetic | Dialysis liquid |
| NVH 0075-95 | Emetic-like | Foodborne outbreak (diarrhoeal |
| NVH 1519-00 | Emetic-like | Foodborne outbreak (diarrhoeal |
| INRA C24 | Emetic-like | Food |
| F3003/73 | Emetic-like | Foodborne outbreak (diarrhoeal) |
| F4429/71 | Emetic-like | Foodborne outbreak (diarrhoeal) |
| NVH 200 | Emetic-like | Foodborne outbreak (diarrhoeal) |
| RIVM BC 63 | Emetic-like | Human feaces |
| ATCC 10987 | Emetic-like | Non lethal dairy isolate |
| WSBC 10892^#^ | Emetic-like | Blood culture |
| WSBC 10028 | Non emetic | Pasteurized milk from Germany |
| WSBC 10035 | Non emetic | Pasteurized milk from Germany |
| ATCC 14579 | Non emetic | *Bacillus cereus* type strain |
| *Bacillus thuringiensis* | | |
| WS 2620 (HER 1236)^+^ | Non emetic |  |
| WS 2621 (HER 1357)^+^ | Non emetic | *Bacillus thuringiensis* subsp*. berliner* |
| WS 2632 (T05001)^+^ | Non emetic | *Bacillus thuringiensis* subsp*. galleriae* |
| WSBC 28001^§^ | Non emetic | *Bacillus thuringiensis* subsp*. kurztaki* |
| WSBC 28002^§^ | Non emetic | *Bacillus thuringiensis* subsp*. kurztaki* |
| WSBC 28022* | Non emetic | *Bacillus thuringiensis* subsp*. tenebrionis* |
| WSBC 28023* | Non emetic | *Bacillus thuringiensis* subsp *kurtzaki* |
| WSBC 28024* | Non emetic | *Bacillus thuringiensis* subsp*. israelensis* |
| *Bacillus mycoides* | | |
| WSBC 10256^‡^ | Non emetic | Soil from Denmark |
| WSBC 10257^‡^ | Non emetic | Soil from Denmark |
| WSBC 10258^‡^ | Non emetic | Soil from Denmark |
| WSBC 10276 | Non emetic | Pasteurized milk from Germany |
| WSBC 10278 | Non emetic | Pasteurized milk from Germany |
| WSBC 10292 | Non emetic | Kurkuma root form Thailand |
| WSBC 10293 | Non emetic | Kurkuma root from Thailand |
| WSBC 10360 | Non emetic | Pasteurized milk form Germany |
| *Bacillus weihenstephanensis* | | |
| WSBC 10001 | Non emetic | Pasteurized milk form Germany |
| WSBC 10045 | Non emetic | Pasteurized milk form Germany |
| WSBC 10202 | Non emetic | Pasteurized milk form Germany |
| WSBC 10204 | Non emetic | Pasteurized milk form Germany, *Bacillus weihenstephanensis* type strain |
| WSBC 10212 | Non emetic | Pasteurized milk form Germany |
| WSBC 10296 | Non emetic | Soil from Germany |

a: INRA: Institut National de Recherche Agronomique, UMR A406, Avignon, France; MHI: Strains from *B. cereus* culture collection at Institute of Hygiene and Technology of Food of Animal Origin, Ludwig-Maximilians-Universität München, Germany; NVH: strains from the Norwegian School of Veterinary Science, Oslo, Norway; RIVM: Rijksinstituut voor Volksgezondheit en Milieu, Bilthoven, Netherlands; UHDAM: University of Helsinki, Department of Applied Chemistry and Microbiology, Helsinki, Finland; WS: Weihenstephan strain collection and WSBC: Weihenstephan *B. cereus* culture collection at Dept. of Biosciences, TU Muenchen, Germany; F-strains were obtained from the Public Health Laboratory Service, London, UK (PHLS).

#Prof. Schmidt, Institut für Medizinische Mikrobiologie und Hygiene, Dresden, Germany.

+H.-W. Ackermann, Université Laval, Quebec, Canada.

§Institute of Microbiology, Wroclaw University, Poland.

*B.M. Hansen, Department of Environmental Chemistry and Microbiology, Rokskilde, Denmark.

‡Damgaard, Copenhagen, Denmark
